# External controls for rare disease drug development: Lessons for emerging and advanced therapeutic modalities

**DOI:** 10.1016/j.omta.2026.201754

**Published:** 2026-05-19

**Authors:** Samuel H. Hughes, Lauren A. Beretich, Matthew Fuller, Kimberly Goodspeed, Melissa Penn, Leonard A. Valentino, Caitlin McCombs

**Affiliations:** 1 Center for Experimental Neurotherapeutics, St. Jude Children’s Research Hospital, Memphis, TN, USA; 2 American Society of Gene and Cell Therapy, Waukesha, WI, USA; 3 Head of Gene Therapy Research, Ultragenyx Pharmaceutical, Somerville, MA, USA; 4 Global Clinical Development, Ultragenyx Pharmaceutical, Novato, CA, USA; 5 Patient Engagement Research and Development, Bayer AG, New York, NY, USA; 6 Hemophilia & Thrombophilia Center, Rush University Medical Center, Chicago, IL, USA; 7 American Society of Gene and Cell Therapy, Waukesha, WI, USA

## Abstract

External controls, particularly natural history studies, play an increasingly important role in rare disease therapeutic development where traditional randomized trials are often infeasible. This review examines regulatory acceptance patterns for gene and cell therapies approved between 2019 and 2025, analyzing successful cases like onasemnogene abeparvovec (Zolgensma) for spinal muscular atrophy and elivaldogene autotemcel (Skysona) for cerebral adrenoleukodystrophy alongside unsuccessful applications. Key success factors include systematic data collection, clinically meaningful endpoints, appropriate patient matching, and disease characteristics that preclude randomization. Recent FDA initiatives, including the Rare Disease Evidence Principles program, signal growing regulatory flexibility, although acceptance remains context-dependent and requires robust data quality standards.

## Introduction

Challenges remain in the development of therapeutics for rare diseases. Beyond limited resources, rare disease drug development faces persistent challenges including modest patient populations, heterogeneous phenotypes, lack of suitable endpoints, and limited commercial returns. These constraints complicate traditional randomized trials and have driven greater use of external controls,^1^ particularly natural history studies that document the untreated disease course, to enhance rare disease therapeutic development.

Natural history studies and other real-world data are not a monolith and can range from patient registries to standardized prospective studies. Therefore, natural history studies are diverse in methodology and rigor, and their use as an external control has led to varied regulatory outcomes.^2^^,^^3^ The US Food and Drug Administration (FDA) has approved drugs using natural history studies as external controls since the 1990s, notably enzyme replacement therapies for Gaucher disease type 1 and hereditary tyrosinemia type 1.^4^^,^^5^ The FDA issued guidance in 2019 to help inform the design and implementation of natural history studies to support the development of safe and effective drugs and biological products for rare diseases.^6^ The types of data that may be considered include retrospective and prospective natural history studies, and data collected from across a cohort of patients during a defined period (cross-sectional study) or for a prolonged period (longitudinal study).

Despite their utility, external controls present several methodological challenges.^7^^,^^8^ Selection bias can arise when treated patients differ from historical comparators in ways that affect therapeutic outcomes. Changes in standard of care can confound comparisons between current treatment cohorts and historical natural history data. Patient heterogeneity can limit comparability. Endpoint drift, where disease assessment methods evolve over time, may introduce systematic differences between historical and contemporary data. Incomplete baseline data or missing prognostic factors can preclude adequate patient matching. These risks reiterate the importance of rigorous natural history study design, prospective data collection when feasible, and careful attention to potential sources of bias.

The FDA’s consideration of external control data as part of the totality of evidence for cell and gene therapy (CGT) approvals to date has had mixed success, due to the mechanism of each therapy, the phenotypic variability of a disease, and the disconnect between FDA guidance and real-world practice. Still, there is continued interest in the development of CGTs from patient advocacy groups (PAGs) who often support natural history studies, along with a commitment from the FDA to support the use of appropriate external controls. The FDA guidance document on drug and biologic development,^2^ Rare Disease Evidence Principles (RDEP) process,^9^ and the newly announced Plausible Mechanism Pathway from FDA^10^ highlight this commitment and the need for updated guidance around the use of external control data in new drug and biologic applications.

Clarity is critical for improved design and use of natural history studies to advance rare disease therapeutics. Here, we discuss learnings from CGT and other foundational programs to provide support for the use of external controls in regulatory decision-making.

## Methods

The approvals presented in Table 1 were identified through a narrative, focused review of official regulatory documentation. This included FDA approval announcements, regulatory summaries, and published literature on rare disease therapeutics approved between 2019 (when FDA guidance was released) and 2025. We focused on approvals where external controls played a substantive role in the regulatory decision, either as the sole comparison or as supporting evidence. This review is not intended to be exhaustive or comprehensive; rather, it highlights representative cases that illustrate key success factors and challenges in external control use.

**Table 1:** Selected rare disease FDA approvals using external controls since 2019

Therapy	Indication	Treatment type	Year approved	FDA rationale for acceptance	Reference
**Retrospective studies**					

Elivaldogene autotemcel (Skysona)	Early, active cerebral adrenoleukodystrophy	*Ex vivo* lentiviral vector hematopoietic stem cell gene therapy	2022	Accelerated approval using MFD-free survival vs. untreated NH cohort.	FDA label; FDA summary basis of regulatory action
Risdiplam (Evrysdi)	SMA	Small-molecule *SMN2* splicing modifier	2020	Single-arm infantile-onset SMA data interpreted against retrospective NH cohorts for survival and motor milestones; placebo-controlled data supported approval for later-onset SMA.	FDA label; FDA summary review

**Cross-sectional studies**					

Eladocagene exuparvovec-tneq (Kebilidi/Upstaza)	AADC deficiency	*In vivo* AAV gene therapy (rAAV2)	2024	Accelerated approval based on week-48 motor milestone achievement compared to untreated NH (intermediate endpoint “reasonably likely” to predict benefit) with PD/biomarker confirmatory evidence.	FDA label; FDA summary basis of regulatory action

**Longitudinal and prospective natural history studies**					

Delandistrogene moxeparvovec-rokl (Elevidys)	DMD	*In vivo* AAV gene therapy	2023	Single-arm trial with pre-treatment baseline motor function data; FDA considered natural history evidence from CINRG Duchenne Natural History Study in evaluation. Accelerated approval based on dystrophin expression (surrogate endpoint); confirmatory clinical benefit trial required	FDA label; FDA Summary basis of regulatory action
Cerliponase alfa (Brineura)	CLN2 Batten disease	ERT	2024	Original approval in symptomatic children (2017) based on placebo-controlled trial. Expanded approval to pre-symptomatic children (2024) based on comparison to natural history controls matched for genotype and age at treatment initiation. Primary endpoint: change in motor-language score on Hamburg scale.	FDA label; Study basis for expanded indication approval
Lonafarnib (Zokinvy)	Progeria, processing-deficient laminopathies	Small-molecule farnesyltransferase inhibitor	2020	Open-label single-arm trial in ultra-rare conditions (Hutchinson-Gilford progeria and processing-deficient progeroid laminopathies). Survival benefit demonstrated through comparison to matched untreated patients from natural history cohorts, accounting for genotype and baseline characteristics. Median survival increase of 2.5 years.	FDA label; FDA integrated review; JAMA survival analysis
Onasemnogene abeparvovec (Zolgensma)	SMA	*In vivo* AAV gene therapy (AAV9)	2019	Survival/motor milestones well characterized historically (two separate NH datasets; PNCR 2014, NeuroNEXT 2017); label and SBRA note NH as comparator.	FDA label; FDA summary basis of regulatory action

**Combination approach**					

Atidarsagene autotemcel (Lenmeldy)	Metachromatic leukodystrophy	*Ex vivo* lentiviral vector hematopoietic stem cell gene therapy	2024	Retro- and prospective NHS pub 2021; clear, large divergence vs. untreated NH on severe motor impairment-free survival and survival; well-characterized disease course enabled NH comparability; FDA SBRA emphasizes unexpected preservation of milestones vs. NH.	FDA label; FDA summary basis of regulatory action;

AADC, aromatic L-amino acid decarboxylase; AAV, adeno-associated virus; DMD, Duchenne muscular dystrophy; ERT, enzyme replacement therapy; NH, natural history; NHS, natural history study; PD, pharmacodynamic; SBRA, summary basis for regulatory action.

The cases presented in Table 1 are drawn exclusively from FDA regulatory decisions. We recognize that evidentiary standards for external controls are an area of active international dialog and that convergence across regulatory agencies will be important as CGTs reach patients globally; a cross-jurisdictional comparison is beyond the scope of this review.

While this review focuses primarily on CGTs, we include select examples from other therapeutic modalities when they established important regulatory precedent for external control use, or illustrate key principles applicable to CGT development.

## Discussion: Regulatory support for external controls

External controls have been assessed by the FDA to support regulatory decision-making for CGTs since the landmark approval of onasemnogene abeparvovec (Zolgensma) for spinal muscular atrophy (SMA) in 2019.^11^ Between 2000 and 2019, retrospective natural history datasets, including retrospective reviews of patient records, were the most common source of external control (44%).^12^ While the FDA guidance emphasizes prospective natural history studies with predefined endpoints,^2^^,^^6^ real-world regulatory decisions demonstrate pragmatic consideration of retrospective data when disease characteristics and data quality meet specific criteria.

Several patterns emerge from programs that effectively used external control data in rare disease CGT development (Table 1). The FDA reviewers appear to favor external controls for degenerative diseases with clearly defined, clinically meaningful outcomes such as motor milestones event-free survival, or mortality.^12^^,^^13^ The acceptance threshold appears to be influenced by disease severity and the ethical feasibility of randomized controlled trials; rapidly progressive, fatal conditions with no available therapies present the strongest case for single-arm trials with external comparators.

Regulatory precedent plays a meaningful role in subsequent approvals. The FDA’s experience with nusinersen (Spinraza),^14^ an antisense oligonucleotide for SMA, established familiarity with SMA’s natural history that facilitated acceptance of external controls for Zolgensma.^15^ Similarly, the Cooperative International Neuromuscular Research Group (CINRG) Duchenne Natural History Study^16^^,^^17^ provided a robust external control framework that supported multiple exon-skipping therapy approvals^18^ and eventually, a gene replacement therapy.^19^ This precedent-building effect highlights the value of well-characterized longitudinal disease registries.

However, regulatory acceptance remains context dependent. Variability exists not only across disease areas but also between FDA review teams and individual reviewers, reflecting the complexity of assessing external controls on a case-by-case basis.^20^ This human element also provides flexibility for approaches tailored to therapeutic contexts.^21^ Moreover, regulatory standards for CGTs continue to evolve as agency experience grows, unlike comparatively well-established modalities such as monoclonal antibodies or small-molecule drugs. Understanding historical acceptance patterns can inform strategic trial design and proactive engagement with the FDA, even when universal rules remain elusive (Table 1).

## Discussion: Selected case studies

The approval of Zolgensma to treat SMA in 2019 represents a paradigm-shifting case of the FDA allowing an external control comparison to a single-arm gene therapy trial. The FDA considered outcomes from an open-label trial in the context of two independent prospective natural history cohorts^22^: the Pediatric Neuromuscular Clinical Research (PNCR) Network and the NeuroNEXT SMA Infant Biomarker Study.^23^^,^^24^ Both cohorts documented median age at death or the requirement for permanent ventilation at approximately 8–14 months in untreated infants with two *SMN2* copies, establishing a clear natural history benchmark. The PNCR study, notably funded by the PAG Spinal Muscular Atrophy Foundation,^25^ demonstrated early stakeholder engagement in building regulatory-grade natural history infrastructure. The FDA’s determination was influenced by multiple factors: the devastating natural history (median survival <1 year), large observed treatment effect (in the pivotal comparison, 91% of treated patients [20 of 22] in the STR1VE-US study were alive without permanent ventilation at 13.6 months, compared to 26.1% [6 of 23] in the PNCR natural history cohort), prospective data collection with predefined endpoints, and independent confirmation from two separate cohorts.^15^ This case illustrates the gold standard when resources and disease characteristics permit prospective natural history studies.

Elivaldogene autotemcel (Skysona) for early active cerebral adrenoleukodystrophy (CALD) demonstrates use of retrospective external control data as supportive evidence for accelerated approval.^26^ The FDA granted accelerated approval in 2022 based on an intermediate clinical endpoint reasonably likely to predict clinical benefit, with contextualization using the ALD-101 natural history study, a retrospective analysis of medical records from children with gadolinium-enhancing CALD lesions.^27^ The retrospective cohort included both untreated patients and those who received allogeneic hematopoietic stem cell transplantation, establishing a 50% major functional disability (MFD)-free survival benchmark at 24 months. A post hoc analysis showed 72% MFD-free survival among Skysona-treated patients versus 43% in matched natural history controls.^28^ The FDA’s decision reflects several mitigating factors: CALD is rapidly progressive and fatal, with approximately 30%–50% mortality within 5 years of symptom onset, randomization to placebo was deemed unethical, and the disease trajectory is well-characterized and predictable. However, this approval was granted under the accelerated approval pathway requiring post-marketing studies,^29^ and subsequent safety events, including 10 reported cases of hematologic malignancy (10/67 treated patients as of August 2025), emphasize both the importance of ongoing monitoring and the risk-benefit considerations inherent in accelerated approval.^30^

Valoctocogene roxaparvovec (Roctavian) for hemophilia A illustrates a related, but distinct, regulatory path worth examining.^31^ Unlike Zolgensma and Skysona, the pivotal study for Roctavian did not rely on an external control cohort; instead, it compared treated patients’ annualized bleeding rates (ABR) to their own within-patient baseline or historical ABR, a standard endpoint previously accepted for hemophilia therapies.^32^ We include this case not as a true external control precedent, but because it demonstrates how FDA scrutiny of comparator adequacy applies broadly across CGT development, regardless of the comparator type. While the FDA did not raise substantive concerns about the use of a within-patient baseline comparator design, the Agency ultimately concluded that available data were insufficient to demonstrate durable clinical benefit, citing uncertainty around the long-term persistence of factor VIII expression.^33^^,^^34^ The FDA required extended follow-up data demonstrating durable factor VIII expression and continued reduction in ABR before granting approval in June 2023, with approval ultimately based on non-inferiority of ABR compared to baseline; factor VIII activity served as a supportive surrogate rather than a primary endpoint.^35^ This case reinforces a theme relevant to external control strategies more broadly: the FDA may require additional evidence of durability even when the comparator approach is accepted in principle, and proactive regulatory engagement remains essential to anticipating and resolving such evidentiary gaps.

Resamirigene bilparvovec (AT132) for X-linked myotubular myopathy illustrates the complex interplay between external control design and overall program viability. The ASPIRO trial used a hybrid control group consisting of 12 natural history participants and 2 concurrent untreated participants to evaluate the treatment’s impact on 24 children, demonstrating statistically significant reductions in ventilator support at 24 weeks.^36^^,^^37^ Importantly, the external control was used to contextualize these efficacy findings and was not identified as a limitation of the trial design. Despite these preliminary efficacy signals, development was halted following three patient deaths, leading to FDA clinical holds.^38^^,^^39^ These safety events, rather than any shortcomings of the external control approach, were the primary drivers of program discontinuation. The AT132 experience demonstrates that even promising comparator approaches cannot overcome fundamental safety concerns and emphasizes the importance of comprehensive risk management in CGT development.

These cases highlight a critical asymmetry in regulatory decision-making: when therapies succeed, external controls often play a direct enabling role in approval, as seen with Zolgensma and atidarsagene autotemcel (Lenmeldy).^40^ However, when programs fail, the reasons are typically multifactorial—encompassing safety concerns, risk-benefit calculations, and trial execution—making it difficult to isolate external control methodology as the primary reason for denial.

## Discussion: Applications for future therapies

Cross-case comparison of external controls reveals several critical considerations for CGT development programs.^41^ First, data quality consistently outweighs study design. Systematic data collection, clear endpoint definitions, and independent validation prove more important than whether data were collected prospectively or retrospectively. Second, disease characteristics strongly influence acceptability. Conditions with predictable, severe, rapidly progressive trajectories and limited therapeutic alternatives provide the strongest cases. Third, when external controls are considered adequate, they are typically one component of a broader evidentiary package including biomarker data, supportive secondary endpoints, and mechanistic rationale. Thus, the FDA’s “totality of evidence” framework is key, rather than sole reliance on historical comparison.^3^ Finally, the rejection of new therapies often stems from insufficient clinical efficacy or safety concerns rather than external control methodology. However, inadequate control data may contribute to the inability to demonstrate treatment effect.^20^

The regulatory landscape is evolving rapidly, and early engagement with the FDA remains essential. Sponsors should present external control proposals during pre-IND meetings, explicitly address potential sources of bias, and propose mitigation strategies. Leveraging regulatory precedent by referencing similar diseases or accepted methodologies can strengthen proposals, although sponsors should recognize that precedent provides guidance rather than guarantee. The case-by-case nature of external control assessment also creates opportunities for tailored approaches that consider unique disease biology, available data infrastructure, and therapeutic mechanisms.

In September 2025, the FDA announced the RDEP program, which provides an expedited pathway for ultra-rare genetic diseases (typically fewer than 1,000 affected individuals in the United States) with significant unmet medical need.^9^ The RDEP program explicitly acknowledges that traditional placebo-controlled trials are often impossible for these conditions and signals openness to single-arm trials with supplemental evidence, including case reports, natural history data, and biomarkers. While details remain limited, the RDEP program represents a meaningful policy shift toward regulatory flexibility for the most challenging rare disease populations. The program’s emphasis on “totality of evidence” and mechanistic plausibility^21^ suggests an expanded opportunity^42^ for innovative trial designs. However, RDEP remains experimental and is narrowly applicable to ultra-rare genetic diseases. Success still requires early engagement with FDA and prospective planning to ensure data collection meets regulatory standards from the outset.

Sustained PAG involvement is essential to direct research priorities and drives the systematic data collection needed to build stronger cases for regulatory acceptance of external controls, ultimately ensuring that CGTs can be developed and approved for rare disease populations. PAGs are increasingly data conveners and methodological partners, co-developing natural history study protocols, endpoints, and long-term follow-up plans. Their community-anchored governance models often enable multi-sponsor participation and sustained, longitudinal datasets. As real-world evidence becomes central to regulatory decision-making, PAGs will remain key enablers of data quality and long-term evidence generation.

## Conclusion

The evolving acceptance of external controls reflects a broader movement toward integrating real-world evidence into regulatory decision-making for rare diseases. While challenges remain, recent FDA initiatives signal commitment to flexibility when scientific rigor can be maintained. The examples reviewed here demonstrate that external controls can be valid comparators when disease characteristics, ethical considerations, and data quality align.

Looking forward, collaboration between PAGs, researchers, industry sponsors, and regulators will be critical to advancing methodological standards and expanding appropriate use of external controls. Well-designed natural history studies, maintained over time with rigorous data governance, represent valuable public goods that can accelerate multiple therapeutic programs and provide meaningful context for evaluating treatment effects in rare diseases where traditional trial designs are infeasible. As the FDA implements RDEP and other initiatives, the rare disease community has an opportunity to shape best practices that balance regulatory rigor with the urgent need to bring safe, effective therapies to patients who currently have no options.

## Acknowledgments

Conceptualization and oversight provided by ASGCT’s Patient Outreach Committee. Administrative support was provided by ASGCT’s Director of Outreach, Alison Kujawski. The article has no funding sources to disclose.

## Author contributions

Conceptualization and writing – review and editing, L.A.B., S.H.H., and C.M.; review and editing, M.F., K.G., M.P., and L.A.V.; project administration, C.M.

## Declaration of interests

L.A.B. was a full-time employee of The Medical Affairs Company, contracted to Intellia Therapeutics, at the time this work was conducted; L.A.B. is now a full-time employee of Intellia Therapeutics. M.F. and K.G. are employees of Ultragenyx Pharmaceuticals. S.H.H. is an employee of St. Jude Children’s Research Hospital. C.M. is an employee of the American Society of Gene and Cell Therapy. M.P. is an employee of Bayer. L.A.V. is a Professor of Pediatrics at Rush University Medical Center and received honoraria and travel support from Regeneron Pharmaceuticals, Inc. for scientific presentations at conferences and also received consulting fees from Inovio Pharmaceuticals, Inc; Novartis Pharmaceuticals, Inc; Regeneron Pharmaceuticals, Inc; Sanofi; and Takeda Pharmaceuticals, Inc. L.A.V. is the Editor-In-Chief, Expert Review of Hematology, and an Entrepreneur-In-Residence for the Pathway to Cures Venture Fund of the National Bleeding Disorders Foundation.
